# Unpacking the Global Rice Trade Network: Centrality, Structural Holes, and the Nexus of Food Insecurity

**DOI:** 10.3390/foods13040604

**Published:** 2024-02-16

**Authors:** Junjie Li, Qin Xiao, Haixia Wu, Jianping Li

**Affiliations:** 1 Institute of Agricultural Resources and Regional Planning, Chinese Academy of Agricultural Sciences, Beijing 100081, China; lijunjie@caas.cn (J.L.); xiaoqin@caas.cn (Q.X.); wuhaixia@caas.cn (H.W.); 2 State Key Laboratory of Efficient Utilization of Arid and Semi-Arid Arable Land in Northern China, The Institute of Agricultural Resources and Regional Planning, Chinese Academy of Agricultural Sciences, Beijing 100081, China

**Keywords:** food security, structural advantages, constraint, global trade, network analysis, rice

## Abstract

The challenging international landscape and exacerbated extreme weather conditions contribute to the instability of global grain trade, complicating its impact on food security. This complexity is particularly pronounced for varieties like rice, which are heavily affected by policy-driven trade restrictions. There is insufficient research on how a country’s rice trade characteristics affect food security. A network analysis approach is adopted to intricately dissect the structural characteristics of rice trade. To explore causality with food insecurity, this paper chooses structural holes and centrality as representatives of trade network characteristics and regresses them on the food insecurity indicator. With cross-national data spanning over 30 years, the network analysis provides a clear portrayal of the dynamic changes in international rice trade. The overall resilience of the trade network has increased, but specific countries’ vulnerability has also risen. Unlike the changing trends in features observed in grain and food trade networks, there is a notable intensification in the imbalance of power distribution in the rice trade network compared to over 30 years ago. The panel data regression results show that constraint, indicating the scarcity of structural holes or connections to stronger trading partners, significantly and positively influences a country’s level of food insecurity. Based on these findings, the policy proposal for importing countries emphasizes creating strategic trade connections. By choosing appropriate trade partners that reduce constraint, food security can be enhanced, even without improvements in other conditions.

## 1. Introduction

Rice is pivotal for global food security, acting as a primary calorie source for millions in Asian poverty and emerging as a significant staple in Latin America and Africa [1]. Compared to the more diversified global wheat and maize markets, the international rice market has a more concentrated production distribution and a smaller proportion of international trade volume [2]. Notably, Asian rice production contributes approximately 90% of the global total, with China and India together contributing approximately 50%. The geographic concentration of rice production makes global output more vulnerable to factors such as the Asian monsoon climate and geopolitical influences, leading to fluctuations, thus making it one of the sources of food insecurity [3,4]. Approximately 40% of Asian rice is produced under a variable monsoon climate [5], leading to substantial interannual fluctuations in rice production that directly impact the supply of the world rice market. Adding to this complexity, climate change is emerging as a critical factor disrupting international trade in the 21st century [6]. The sensitivity of rice to climate change implies that its production is significantly affected by climatic variations, such as floods, droughts, and salinization. This sensitivity becomes particularly crucial when considering the historical role of the global rice market in previous food crises, notably the 2008 global rice crisis when several importing countries faced severe rice shortages.

While production serves as the foundational factor determining supply, trade fluctuations often emerge as a significant source of food crises, especially in the case of rice. Many Asian countries ascribe cultural and political significance to rice, considering it a signal of food security [7]. This prioritization makes rice the most protected grain and contributes to greater instability in its international trade compared to other grains. Since the Russia-Ukraine conflict, as of 5 June 2023, India, Bangladesh, Cameroon, Kuwait, Lebanon, and Russia have implemented export bans on rice. Exploring innovative trade strategies is essential to elevate the food security levels of importing countries. Considering these complexities surrounding the global rice market and its impact on food security, it becomes crucial to recognize the multifaceted role that trade plays in shaping the dynamics of food security. In scenarios where trade relationships are stable, countries can confidently rely on external supplies, leveraging comparative advantage rather than resorting to costlier domestic production without a comparative advantage. However, when the international trade market becomes unstable, dependence on imports becomes a source of risk for food security [8]. Previous research, primarily measuring vulnerability based on factors such as dependency on foreign sources [9], underscores the intricate relationship between trade and food security. Studies on the international rice trade underscore the high vulnerability of importing countries and the considerable influence of large exporting nations. Many rice-importing countries, such as Saudi Arabia, South Africa, and Belgium, lack domestic production resources. The absence of a stable external supply can significantly impact their food security [10,11]. Some researchers narrow their evaluation of importing country vulnerability to indicators such as the external dependence index and import concentration index, focusing on production fluctuations rather than trade fluctuations on the exporting side [8,12,13].

A social network analysis begins with examining relationships by delving into network structures and central positions. This approach offers a detailed and precise measurement of trade structural characteristics, providing multiple perspectives on the sources of power status in trade. By studying the characteristics of trade networks and their implications for food security or vulnerability, one can gain new insights for explaining food crises and identifying potential solutions. The impact of food production shortages on food supply is not solely determined by global markets; underlying trade networks also play a crucial role [14]. The increasing connectivity and mobility of global trade networks, coupled with the growing trend of food import dependence, heightens the vulnerability of food systems to systemic disruptions [15]. Furthermore, trade networks can trigger cascading effects, potentially transforming localized shocks into global crises [16]. Notably, the rice trade has been identified as being particularly susceptible to cascading export restrictions, with Asian and African countries being the most exposed to such cascades. An analysis of the trade structure enables the identification of primary sources of constraints and provides pathways for strategy improvements [17].

The primary network characteristics examined in the literature include centrality, small groups, and network density [17,18], with fewer studies focusing on the analysis of structural holes. In this context, analytical models predominantly utilize statistical descriptive analysis [10], the construction of risk indicators [8], impact simulation [19], and the multilevel impacts of shocks [16], with less emphasis on causal analysis. These research findings generally show the increased activity and closeness of international trade, while diverse conclusions have been drawn regarding the impact of shocks on food security from different perspectives [8,10,16,19].

Importation is identified as a significant source of instability in food supply, particularly with rice imports causing more widespread instability in food supply regions than corn and wheat imports. The reasons for instability caused by rice imports vary across different regions, emphasizing the need for a regional heterogeneity analysis [10]. Structural hole analysis is more prevalent in the field of business organizations than in the context of trade networks; however, the existing literature consistently highlights the positive impact of occupying structural holes on organizational outcomes, underscoring the strategic opportunities available to organizations aware of their advantageous positions [20]. One can assume that occupying structural holes in the rice trade network could impact a country’s food security. A recurring issue in the literature revolves around the extent to which trade network characteristics affect food security, particularly the challenge of establishing causality [21,22].

Existing research predominantly focuses on the resilience revealed by trade network characteristics and potential food security risks. However, there is limited exploration of causal analyses regarding how the network structure characteristics of a single country affect its level of food security. The degree to which network characteristics influence food security remains a complex and unresolved issue in the literature. Despite adopting the recommended strategy of import diversification, some countries still face the risk of unstable imports, leading to domestic food shortages. A potential explanation for this phenomenon can be found in the structural features of their import partner relationships. This paper innovatively utilizes computed panel data on network features as explanatory variables, offering potentially deeper insight into these complex relationships.

This research endeavored to bridge existing gaps in understanding the evolving nature of international rice trade and its impact on food security. The main problem addressed in this study is the extent to which the trade network structure characteristics of a country affect its food security. The primary goal of this study is to identify the sources of food insecurity, the origins of vulnerability, and the specific areas that can be fortified. Drawing on 36 years of trade matrix data for 210 countries, the authors calculated and analyzed the dynamics of dozens of indicators measuring the characteristics of the international rice trade network. To test the effect of centrality and structural holes on a country’s level of food insecurity, a regression model was developed based on the panel data. The results have the potential to deepen the understanding of the role of trade networks and provide a scientific basis for policies aimed at optimizing networks to enhance food security. This study holds immense significance in unraveling the intricate dynamics of the international rice trade network and its profound implications for global food security.

The upcoming chapter is organized as follows. The second and third sections elucidate the calculation formulas of the main indicators used in the network analysis and describe a panel regression model constructed based on core indicators, providing detailed insights into the sources and processing procedures of the primary data. The fourth section illustrates the dynamic changes of network analysis indicators along with the regression results of centrality and constraint indicators on the level of food insecurity. The fifth and sixth sections discuss the findings and provide concluding remarks.

## 2. Methods and Data

### 2.1. Trade Network Analysis

For the social network analysis, trading countries or regions are designated as nodes, and the trade flow between them are considered edges, thus forming a complex trade network [23]. This network is succinctly represented by a square matrix, *W_n_*, with dimensions of n*n, where exporters are in the rows and importers are in columns. To comprehensively investigate the complex network characteristics of the international rice trade, a weighted trade network (*W_n_*) based on value data and an unweighted trade network (*A_n_*) derived from *W_n_* are constructed. The unweighted network (*A_n_*) is generated by disregarding the link weights and solely accounting for the presence of a trade connection. The analysis employs both network-level and node-level measures to provide a comprehensive understanding of the international rice trade network. The square matrix *W_n_* can be represented as

(1) $$W_{n}^{t}=\left(\begin{array}{ccc} w_{11} & \cdots & w_{1n}\\ \vdots & \ddots & \vdots \\ w_{n1} & \cdots & w_{nn} \end{array}\right)$$

where *t* represents the year, and the matrix elements, *w_ij_*, represent the trade volume between node *i* and *j*. In the square matrix, *A_n_*, the matrix elements are either one when there is a trade connection or zero when there is no trade connection between the two nodes.

Table 1 lists more than 30 measures of network-level features [24,25,26]. The measures are categorized into three main groups: network size, network centralization, and connectivity. The measures of the network size mainly reveal the trade connections and volume. The network centralization indicators generally reflect the degree of imbalance in the distribution of trade connections or trade volume among countries. The network connectivity’s main focus is to reflect the efficiency of trade connections, local fragmentation, and reciprocity.

Within the node-level measures, centrality and structural holes are explored. Centrality stands out as one of the most fundamental and crucial concepts in network analysis, providing insights into the superiority or privilege of a node within the network. There are various methods for measuring centrality, the most basic of which is the node degree. In an unweighted network, the node degree (*k_i_*) represents the number of connections a node has, reflecting the number of countries with which a particular country trade. Mathematically, the node degree (*k_i_*) is defined as

(2) $$k_{i}=\sum_{j}a_{ij}$$

where *a_ij_* is the element of the binary adjacency matrix, *A_N_*. For weighted networks, the weighted counterpart to degree is the node strength (*s_i_*), calculated as $s_{i=}\sum_{j}w_{ji}$. The degree can further be divided into outdegree (export) and indegree (import) based on the direction of trade. A higher degree is indicative of greater centrality or influence in the network.

The concept of structural holes serves as a foundational explanation that transcends player attributes, populations, and time [27]. This concept captures a causal mechanism through which personal advantages can be derived from network positions [28]. The constraint measure describes the scarcity of structural holes possessed by a node. The value of the constraint is higher when a node has fewer or more mutually strong (i.e., more redundant) contacts [29]. In simpler terms, a node’s constraint is typically greater if the ego network is smaller or if its trading partners are highly connected—either directly in a dense network or indirectly through respective central contacts in a multitiered network. Burt’s measure of constraint, *C_i_*, for vertex *i*’s ego network, *V_i_*, is defined for directed and valued graphs as

(3) $$C_{i}=\sum_{j}{\left(p_{ij}+\sum_{q}p_{iq}p_{qj}\right)}^{2}$$

For a graph of order *n*, the proportional tie strengths are defined as $p_{ij}={(a}_{ij}+a_{ji})/\sum_{j}(a_{ij}+a_{ji})$, where *a* represents the elements of the graph adjacency matrix, *A*. Notably, for isolated vertices, the constraint is undefined [27].

### 2.2. Regression Model

At the network level, indicators are chosen to either align with or counter the trends observed in food security. At the node-level, the focus is on identifying indicators related to the food security index based on the regression model. A panel data model is established to uncover causal relationships. The main model takes the following form:

(4) $$Y_{i,t}=\propto +\beta_{1}{out}_{i,t}+\beta_{2}{in}_{i,t}+\beta_{3}C_{i,t}+\gamma X_{i,t}+\delta_{i}+\mu_{t}+\varepsilon_{i,t}$$

where *i* and *t* represent country and time, respectively. *Y* denotes food insecurity and is represented by the prevalence of undernourishment (PoU), which is an indicator released by the FAO to measure the severity of hunger [30]. Undernourishment, as per the FAO’s definition, occurs when a person’s usual food consumption is insufficient to provide the dietary energy needed to maintain a normal, active, and healthy life.

The key explanatory variables in the model include network centrality indicators and structural hole indicators. The variable *out_i,t_* consists of indicators calculated through network analysis to gauge the centrality of export directions. Similarly, the variable *in_i,t_* comprises indicators that measure the centrality of import directions. Additionally, *C_i,t_* represents the value of constraint, indicating the extent of a country’s lack of structural holes.

The variable *X* constitutes a set of control variables including GDP per capita (lnGDP) and the cereal import dependency ratio (CIDR), which are hypothesized to be significant determinants of national food security [31,32]. The term $\varepsilon_{i,t}$ represents a random disturbance term following a standard distribution. $\delta_{i}$ denotes country-specific effects, encapsulating factors such as geographic characteristics or enduring cultural and institutional elements that remain relatively constant over time. Additionally, $\mu_{t}$ represents the time-specific effect, accounting for factors such as fluctuations in world prices and addressing shocks that universally affect all countries, such as global demand shocks. In the regression model, period-fixed results are presented because global food shocks have a broad impact on most countries, and fixing this variable helps control for such effects [33,34].

### 2.3. Data

The trade data spanning from 1986 to 2021 were sourced from the detailed trade matrix dataset of the Food and Agriculture Organization Corporate Statistical Database (FAOSTAT) (http://www.fao.org/faostat/en/#home, accessed on 2 July 2023) [35]. FAOSTAT provides extensive global coverage and longitudinal data, enabling researchers to monitor trends over time across diverse countries. Although the credibility is reinforced by contributions from official departments of many countries, there is a potential for inaccuracies, especially when certain nations have limited capacity for comprehensive data collection and reporting. In particular, countries with lower economic levels are more likely to have missing data, leading to sample bias. Specifically, the data for imports and exports are adopted using the item classification of rice/paddy (milled rice equivalent). The export quantity is used to construct the main network. In instances where country-specific data are unavailable, they are supplemented by the conversion of import data. There are slight discrepancies between the sum of all countries in our matrix and the world rice trade volume. However, the difference between the two datasets for the last 10 years is negligible, amounting to less than one percent. Although the disparity was more pronounced in earlier years, it did not significantly impact this study of trends and changes. This study’s trade matrix database comprehensively covers all countries or regions engaged in the rice trade from 1986 to 2021. In cases where a country’s name changed, the pre- and post-change names are treated as separate nodes to maintain consistency. The number of nodes is consistently set at 210 for comparability across years, even though the actual number of countries or regions engaged in trade each year was generally slightly less than 210.

The PoU, GDP per capita, and the cereal import dependency ratio are sourced from the suite of food security indicators and the macrostatistics dataset of FAOSTAT (http://www.fao.org/faostat/en/#home, accessed on 2 July 2023) [35]. The panel data utilized in the base regression model include 180 countries spanning from 2001 to 2021, representing approximately 99% of the total world rice trade volume. The PoU is presented as a three-year average for intermediate years, and missing data in the cereal import dependency ratio are imputed using the average of three neighboring points.

Notably, the PoU measures levels of food insecurity, with a smaller value signifying greater food security. The global prevalence of undernourishment decreased from 2002 to 2014, slightly increased in 2015, and then declined again. However, after reaching a low point of 7.5% in 2017, the trend reversed and it reached 9.3% in 2021, essentially returning to the 2009 level. Global Food Security Index (GFSI) values for the robustness check (2012 to 2021) were obtained from The Economist website (https://impact.economist.com/, accessed on 21 July 2023). This study employs Ucinet 6.750 for network analysis and Gephi for visualization purposes (https://gephi.org/, accessed on 15 August 2023) [25].

## 3. Results

### 3.1. Network-Level Structure and Dynamics of the Global Rice Trade Network

To better understand the multifaceted factors influencing food security, the first step is to determine the network-level structure and dynamics of the global rice trade network using social network analysis techniques. Table 2 illustrates the alterations in the values of key network-level characteristics. The global rice trade network underwent substantial expansion over the past thirty years, with 2149 trade connections in 2021—four times more than in 1986—among the 210 nodes. The density of the network grew grown from 1.00% to 4.90%. The whole network became tighter and more cohesive. The number of components and component ratios decreased, suggesting fewer disconnected subgraphs in the network. An increase in compactness from 4.5% to 20.8% suggests that nodes in the network became more closely connected and may indicate the formation of tighter clusters or communities within the network. On average, all nodes became closer to each other, as evidenced by the increase in values of connectedness and transitivity. The average distance and SD distance became shorter, signifying a higher level of trade transport efficiency within the network. The network-level analysis revealed substantial increases in size, density, and efficiency, signifying enhanced cohesion and resilience across the entire rice trade network. However, the overall resilience relies on some core nodes, and their influence may be substantial.

Centralization imbalances continued to rise, especially in the export direction. Between 1986 and 2021, out-centralization, representing overall network centrality in the export direction, surged from 23.10% to 73.40%, indicating a significant increase in the power disparity driven by exports. The indeg h-index rose from 8 to 20, aligning with the expanded network size and signifying that more countries within the trade network gained a substantial number of trading partners. Simultaneously, the in-centralization recorded at 17.2% in 2021 reflects a relatively decentralized power distribution. The K-core index increased from 7 to 18, indicating a growing centralization of the network around highly connected nodes and enhanced resilience against random node removal. However, the increase in trade flows on the largest edge (maximum strength) is much greater than the increase in average flows (avg strength), partly reflecting a growth in imbalances.

Despite a 12.2 percentage point increase in transitivity/closure, this figure does not match the substantial increase of 32 percentage points in connectedness. The transitivity/closure value in 2021 implies that the remaining 65.3% of the population must be connected through intermediary nodes to reach a third party. Coupled with a twofold increase in the dependency sum, this suggests that more bridges or intermediary nodes gained significance, leading to increased dependency on them. Link reciprocity remained low. In 2021, 91.2% of node pairs had no links (nulls), 1.1% had mutual links (mutuals), and 7.7% had unreciprocated links (asymmetrics). Arc reciprocity, the proportion of existing bidirectional connections, was 21.9%, and dyad reciprocity, the bidirectional connection proportion among connected pairs of nodes, was 12.3%. Although both arc reciprocity and dyad reciprocity increased after 1986, the proportion of such cases remained relatively small, indicating the presence of substantial heterogeneity and structural holes in trade connections.

**Figure 1:**
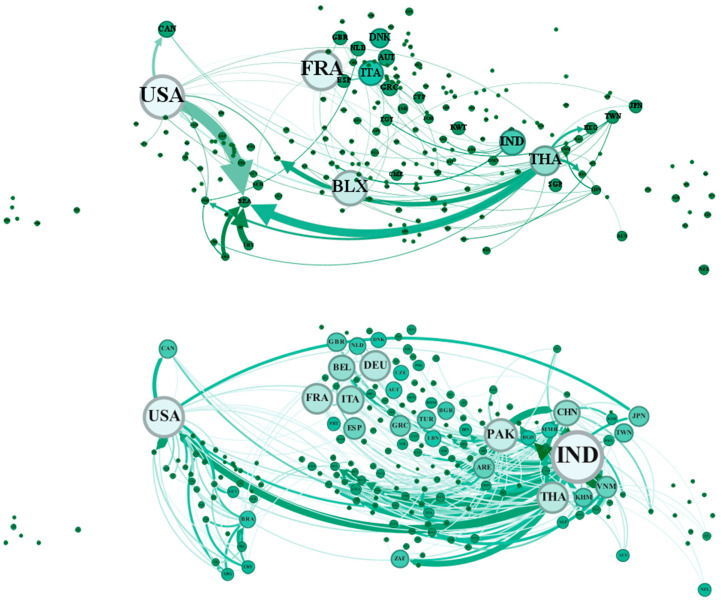
Visualization of the global rice trade network in 1986 (**top**) and 2021 (**bottom**). The sizes of nodes and edges in the graph reflect only their relative magnitudes within each graph.

Figure 1 visually represents the nodes and flow of the rice trade network. The node size reflects the node strength, which represents the volume of the rice trade, while the edge thickness corresponds to the trade volume of specific connections. For readability, both graphs include edges with quantities exceeding 10,000 tons. In the 2021 graph, there are 358 such edges, comprising 16.66% of the total edges. In the 1986 graph, only 83 edges are displayed, accounting for 19.48% of the total. The graphs reveal an approximate trend toward increased density and efficiency in the network. The United States of America (USA), India (IND), and Thailand (THA) maintained their central export positions, with India emerging as the most important exporter. France (FRA) and Belgium-Luxembourg (BLX) that were core trade nodes in the rice trade in 1986 experienced significant decreases by 2021. The country codes are listed in Table A1.

The network-level analysis highlights increased density and efficiency trends, emphasizing the evolving dynamics and key players in the global rice trade network. The global rice trade network became more cohesive, with fewer disconnected subgraphs and the formation of tighter clusters. While overall resilience improved, centralization imbalances surged, particularly in the export direction.

### 3.2. Node-Level Distribution of Centrality and Structural Holes

While network-level measurements provide a holistic view of the whole network’s structure and characteristics, node-level measurements offer insights into the importance and role of individual nodes within the network. The centrality and structural holes endow nodes with structural advantages, making them significant sources of power dynamics in trade. Hence, they are key indicators in the analysis. Node centrality can be measured by degree, closeness, and betweenness centrality. Each measure has its own definition of importance and power source. Degree centrality assigns an importance score based simply on the number of links held by each node, as calculated by Equation (2). In a weighted graph, importance can be assigned based on the flow between the ties, as represented by strength in the analysis to distinguish it from degree. Closeness centrality measures the ability of a node to reach others along a shorter path, occupy a better position in the network, and have a structural advantage that can be converted into power. Betweenness centrality, being in the middle of other nodes, brings structural advantages. The more connections there are in the middle, the less dependence there is and hence, the more power there is. To reflect the dynamic changes over the past 30 years through a few typical points, structural trend changes are identified in 1992, 2008, and 2014 based on multiple break point tests and trend observations of the time series data on global rice trade volumes. Since 1992, the trade volume-to-production ratio for rice has increased due to the relaxation of trade barriers among countries [36]. The structural changes in 2008 were primarily driven by the global food crisis in 2007–2008, leading rice-exporting countries to impose export bans, significantly reducing liquidity in the international rice market [37]. The change from 2014 is mainly due to the increase of trade volume based on record imports by Sub-Saharan Africa and China, slightly lower global trading prices and abundant exportable supplies [38]. Incorporating the start and end years, the data from these five years are utilized when presenting revolution patterns. Table 3 provides a listing of the top five countries based on degree centrality, closeness centrality, and betweenness centrality.

In more recent years (2008, 2014, and 2021), India has consistently held the top position in terms of outdegree, outstrength, and outcloseness centrality. While India’s export volume surpassed that of Thailand only in the last decade and India has become the leading exporting country, it was ranked first in terms of outdegree and outcloseness centrality in 2008. In 2021, India’s ego network comprised 164 nodes, including itself, making up 78.1% of all network nodes. India exports to all 163 countries, with the top five—Bangladesh (BGD), Benin (BEN), China (CHN), Senegal (SEN), and Nepal (NPL)—each receiving exports exceeding one million tons. In terms of centrality measured by betweenness, the United States has consistently held the top position. In 2021, the United States’ ego network comprised 122 nodes, representing 58.1% of all network nodes. USA exports to 101 countries and imports from 47 countries. Germany (DEU), France (FAR), and South Africa (ZAF) ranked in the top five for betweenness centrality in 2021. Despite being net-importing countries, they also act as import sources for numerous other nations.

Another crucial indicator of a node, aside from centrality, is its structural holes, as calculated by Equation (3). Structural holes identify countries with greater power due to a particular structural advantage, while a constraint serves as its counterpart, explaining why countries have greater vulnerability due to limitations stemming from structural disadvantages. A constraint is a cumulative numerical value that represents the degree to which the ego is connected to other interconnected entities. If the potential transaction partners of the ego are also potential partners with each other, the ego faces greater constraint. Conversely, if the ego’s partners have limited alternative choices, they cannot constrain the ego’s behavior. The concept of constraint highlights that actors with numerous connections may lose autonomy rather than gain more freedom, and the balance of losses and gains is determined by the relationships among the actors [25,27]. For instance, in 2021, China (CHN) had the highest import volume, thus ranking first in terms of volume. However, it had only 15 import sources, thus ranking 38th in terms of sources. Despite this, China exhibited relatively low constraint. This is primarily attributed to the fact that its partner countries have fewer trade connections, and there is limited interaction among these partner countries.

Dyadic constraint is a measure in network analysis that assesses the level of constraint in a node pair’s relationships within a network. For instance, in a scenario where node A has few other ties except that to B and where A’s other connections are also linked to B, A would experience a higher level of constraint from B [25]. From the perspective of food security, the focus is mainly on the constraint imposed by the import direction. For example, India is a major import source for many countries, and these countries connected with India have few independent connections other than India. There are too few alternative options, and even if there are other connections, they are also countries that import from India. Hence, India has a substantial influence on the constraint faced by these nations. According to the 2021 import direction data, the five countries with the most significant constraint on other countries were India (IND), China (CHN), the United States of America (USA), Thailand (THA), and Pakistan (PAK). Countries experiencing significant high constraint imposed by India are primarily located in Asia and Africa, with the top five nations being Bhutan (BTN), Liberia (LBR), Chad (TCD), Yemen (YEM), and Nepal (NPL). The countries facing substantial constraint imposed by China are predominantly located in Oceania and Africa. The countries subjected to significant constraint imposed by the United States are primarily located in Latin America and the Caribbean, with the top five nations located in Central America and the Caribbean. The top countries facing the highest constraint imposed by Thailand include New Caledonia (NCL) and French Polynesia (PYF) in Oceania. The 10 countries facing the highest constraint imposed by Pakistan include nations in Asia and Africa. At the same time, Pakistan faces significant constraint from China and India.

In comparison with major exporting countries, Vietnam imposes relatively lower constraint on other countries, while China has relatively more constraint on other countries. Among the major importing countries in 2021, China experiences light constraint, primarily from Vietnam with a dyadic constraint value of 0.175. Bangladesh faces significant constraint, mainly imposed by India, with a constraint value of 0.923. Benin is primarily constrained by India, with a constraint value of 0.639. The United States encounters constraint to a certain extent, primarily imposed by Thailand with a constraint value of 0.268.

**Figure 2:**
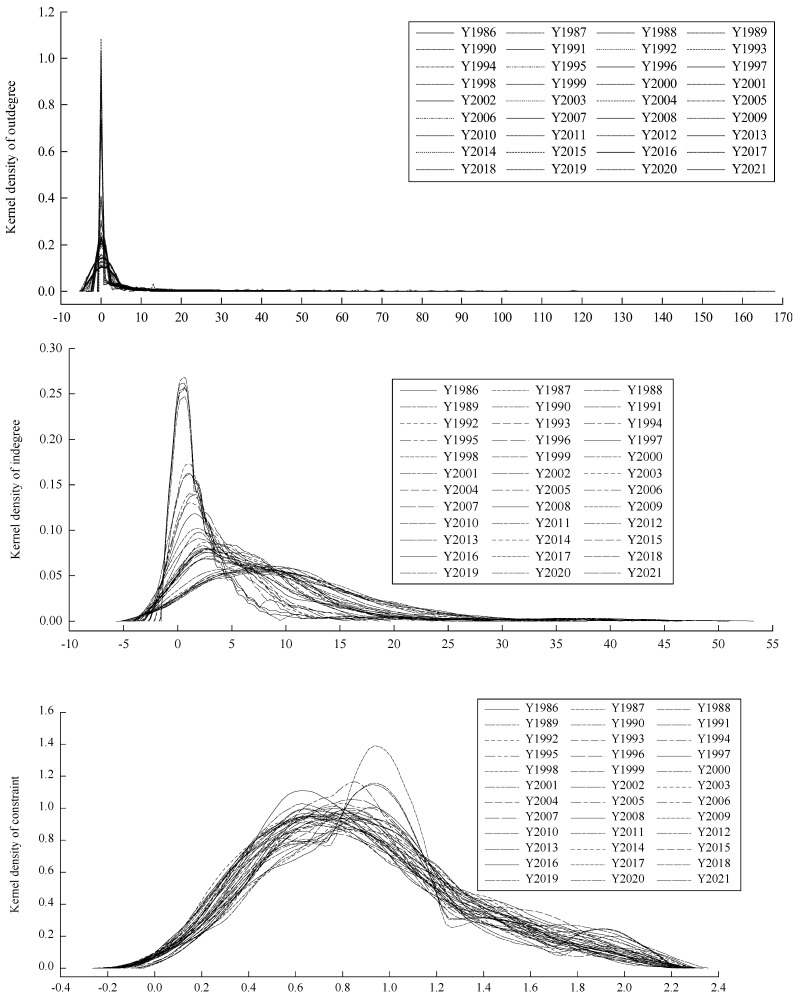
Kernel density distribution of outdegree, indegree and constraint from 1986 to 2021.

Figure 2 shows that the kernel density plot for outdegree is concentrated around the value of 0, signifying that the majority of countries have relatively few connections in terms of rice exports. Over time, the peak of the kernel density exhibits significant fluctuations. After reaching its highest point at 1.03 in 1991, the peak value demonstrates a continuous decreasing trend, reaching 0.107 in 2021. This implies that overall, the number of countries with very few or no trade export connections has decreased over the past three decades. The peak value of the kernel density curve for indegree countries (importing countries) has consistently decreased over time, declining from 0.26 in 1986 to 0.063 in 2021. Additionally, the overall distribution has gradually shifted rightward, signifying an increase in sources of rice imports across many countries over the years. The kernel density distribution of the constraints reveals a distinct leftward shift in the peak value, suggesting a decrease in the number of countries with high constraint values. The peak shows relatively minor changes in general, with values of approximately 1.01 in 1986 and 0.89 in 2021.

The node-level centrality and structural holes provides insights into the significance and role of individual nodes in the global rice trade network. The distribution of power among countries is still highly disproportionate, and many countries remain vulnerable in the global context.

### 3.3. Causal Estimates of Network Variables and Food Insecurity

#### 3.3.1. Estimation of the Main Model

The PoU (*Y*_1_) is used as the dependent variable in the main model. A country’s centrality in the network is measured by multiple indicators, which show slight differences but strong correlations. The outdegree and indegree indicators are chosen to represent node centrality because compared to other sets of centrality indicators, they exhibit smaller numerical differences, provide more reliable data, and are easier to comprehend. Additionally, the constraint variable, representing the degree of absence of structural holes, further refines the network analysis. Control variables, such as the logarithm of GDP per capita (lnGDP) and the cereal import dependency ratio (CIDR), are included to contextualize the broader dynamics within the network.

Firstly, a summary of the descriptive statistics is presented for the variables employed in the main regression model, as represented by Equation (4), aiming to enhance the comprehension of the sample (Table 4). The mean PoU is 9.730, signifying that on average, 9.73% of a country’s population experiences undernourishment. The maximum PoU is 67.800, indicating that during the most challenging year, 67.8% of the country’s population experienced undernourishment. The mean values for outdegree, indegree, and constraint are 9.623, 9.589, and 0.847, respectively. Notably, outdegree exhibits a relatively large standard deviation. The average cereal import dependency ratio (CIDR) implies that, on average, each country needs to import 24.97% of its food supply. A negative CIDR value means that the country is a net exporter of food.

Table 5 shows the regression results of the main model. The focus is on the results in column (6). The adjusted R-squared value indicates that 51.12% of the change in the dependent variable can be explained by the change in the independent variables. The constraint level has a substantial and significant impact on food insecurity. The constraint coefficient, measured at 2.75, significantly surpasses the indegree coefficient. This finding suggests that a one-unit increment in the constraint value corresponds to a 2.75 percentage point increase in the PoU. Thus, a reduction of 0.36 in the constraint value could result in a one percentage point decrease in the PoU.

Regarding another important dependent variable, the impact of import centrality is statistically significant, although the coefficient is relatively small, indicating a modest influence on food security. The coefficient for the indegree is 0.0836, indicating that with other variables held constant, a one-unit increase in its value (reflecting a growth in the number of import connections) is associated with a 0.08 percentage point reduction in the PoU. Put differently, increasing the PoU by one percentage point would require the establishment of approximately 13 new trade connections. The indegree may reflect food security from two perspectives: on the one hand, high import centrality might signify a large volume of imports, potentially but not necessarily implying a lower level of food security; on the other hand, a high import centrality suggests diverse sources of imports, contributing to enhanced food security.

Unlike the import centrality, export centrality appears to exert a relatively minor and insignificant influence on food insecurity. The nonsignificant regression coefficient of the outdegree, coupled with its modest magnitude, suggests that export centrality may not substantially influence PoU. This phenomenon is understandable, especially considering that some prominent rice-exporting nations prioritize foreign exchange accumulation over achieving food self-sufficiency. For instance, India primarily participates in rice exports to bolster foreign exchange reserves, subsequently resorting to the import of other essential food crops such as corn.

As for the control variables, for every 1% increase in per capita GDP, the PoU decreases by 5.28 percentage points. A 1 percentage point increase in the cereal import dependency ratio (CIDR) is associated with an increase of 0.01 percentage points in the PoU.

A comparison of the coefficient values for indegree, CIDR, and constraint reveals that the primary contribution to the PoU does not stem from import dependency but rather from the structural characteristics of import connections. This inference suggests that establishing connections with the less core countries might substantially reduce constraint values, effectively decreasing the level of food insecurity.

#### 3.3.2. Robustness Check and Endogeneity Mitigation

Alternative food security measures are employed to assess the robustness of the regression results. The indicators adopted are the average dietary energy supply adequacy (*Y_e_*) from the FAO database and Global Food Security Index (*Y_GFSI_*) developed by Economist Impact. Based on the model’s adjusted R-squared value, the explanatory variables demonstrate greater explanatory power for *Y_GFSI_*, potentially attributable to the smaller sample size. The GFSI involves only 113 countries, making the sample much smaller than the 180 countries used in the analysis, leading to significant differences between the sample and the main regression model. Despite these disparities, the regression results reveal significant coefficients for both indegree and constraint, which is consistent with the main model (Table 5).

The regression method used is a two-stage least squares (2SLS) approach to address potential endogeneity issues, acknowledging potential correlations with omitted variables in terms of both degree centrality and constraint. The instrumental variables (IV) adopted are the total agricultural trade network’s centrality and constraint measures. These instrumental variables are related to the centrality and constraint indicators in the rice trade network but do not directly impact a country’s food deficiency rate. The first step is to regress instrumental variables and other exogenous variables to estimate endogenous variables. These estimates are then substituted into the original equation to derive regression coefficients. Table 6 shows that the coefficients of Findegree and Fconstraint are positive and significant, which is consistent with the main tests.

#### 3.3.3. Heterogeneity Analysis

Firstly, regional dummy variables representing the five continents are introduced into the main regression equation. The coefficients represent the average impacts of Asia (coefficient of −4.32), Europe (−5.00), the Americas (−3.09), and Oceania (−3.42) on the PoU in comparison to the reference region (Africa). The negative values signify that the average PoU in continents other than Africa is significantly lower than that in Africa. This finding aligns with that of the literature [39], which reports that the proportion of undernourished people in Africa reaches nearly 20%, surpassing that in other global regions. In comparison, this figure was 8.5% in Asia, 6.5% in Latin America and the Caribbean, and 7.0% in Oceania 7.0% in 2022.

Secondly, the sample is divided into distinct groups, and separate regression analyses are conducted for each group to gain a more nuanced understanding of the heterogeneity of the data. The regression analysis is conducted on the subset of the sample, grouping it based on the five continents and considering two subareas: the region of Latin America and the Caribbean and that of Sub-Saharan Africa.

Table 7 presents the regression results for distinct geographical regions. Notably, the constraint coefficient exhibits significant and high values in Asia, Sub-Saharan Africa, Latin America, and the Caribbean. These regions correspond to the prevalent distribution of countries where rice is a staple food. These findings indicate that in regions where rice constitutes a primary dietary component, the influence of the level of constraint on food security is more pronounced than in areas where rice is less prominent in diets. Table 7 also presents the regression results for regions grouped by economic and social indicators. The constraint coefficient exhibits significant and high values in all three groups: least developed countries, low-income food deficit countries, and net food-importing developing countries.

## 4. Discussion

This study conducted an in-depth network analysis of the dynamics within the global rice trade, exploring its connection to food insecurity. This initiative addressed the existing literature’s inadequate attention to enhancing the structural advantages of trade networks. The results encompassed analyses at the network-level and node-level and a regression model analysis using panel data. The main findings were as follows: (1) the rice trade network became more cohesive and resilient as a whole, while relying more on the core countries; (2) the power distribution based on the rice trade network positions was more unequal between countries; (3) furthermore, the occupation of structural holes have a significant impact on food security. Hence, improving the value of structural holes, i.e., establishing connections with partner countries that are less connected or have weaker ties, could improve a country’s food security level.

The network-level analysis revealed substantial increases in size, density, and efficiency, signifying enhanced cohesion and resilience across the entire rice trade network. For example, global rice trade connections have expanded fourfold over the last 30 years. Nevertheless, the overall resilience is dependent on certain central nodes, and their impact may be considerable. The indicators measured were network density, centrality, and connectiveness of the international rice trade over the last three decades. For network-level measures, a general trend observed is the increasing efficiency and complexity in global food trade networks. Higher efficiency was signified by the fourfold expansion of trade ties, higher density, and shorter average distance. Complexity was evident in the generally tighter connections for each node on average, while there was also an increase in the degree of unequal distribution. This trend of more closely connected food trade networks was consistent with the findings of the World Cereal Trade Network from 1986 to 2013 [40], a food trade network analysis from 1992 to 2018 [41], and the International Wheat Trade Network for the period 2009–2013 [19]. However, as noted by the previous literature, the global network has become more resilient overall, but certain developing countries remain vulnerable [19]. This study‘s analysis of the global rice trade network reveals several crucial features that impact food security. Contrary to the suggestion that a more dispersed structure be established for the global food and agriculture trade network [42], this paper’s findings in the rice trade network context indicate that there has been an increased concentration in and dominance of central countries. In 2021, the network exhibited a substantial level of power disparity, with a high whole network centrality in the export direction (73.4%) and a comparatively lower centrality in the import direction (17.2%). This disparity can be visually reflected by the rice export volume proportion of the primary exporting country, which increased from 34.0% in 1986 to 41.5% in the 2021, compared to the world rice export volume. A significant portion of this growth is attributed to the substantial increase in rice exports from India, aiming to boost its foreign exchange reserves [43]. The degree of fragmentation highlights that more than half of the node pairs lack mutual connections, indicating a certain level of isolation among countries. The transitivity/closure and reciprocity measures suggest a significant reliance on intermediary nodes for trade connections, contributing to both structural complexity and potential vulnerabilities in the network.

The node-level analysis highlighted that the distribution of power among countries was still highly disproportionate. India, Thailand, Pakistan, Vietnam, and the United States consistently ranked among the top five influential countries across various dimensions in the rice trade network. These core countries in the rice trade network intersect with key players in other grain markets. The six most central countries in the wheat trade network are Germany, Italy, France, Turkey, Russia, the United States, and Canada [22], while those in the maize market are United States, Argentina, and Brazil [21]. This aligns with Zhang et al.’s observation of stable core exporting countries for various crops as including the United States, Canada, Argentina, Brazil, and India [8]. This study further highlighted countries that play crucial intermediary roles in the rice trade network, like China, Italy, and Greece. These intermediary countries, although not the most important exporters, could exert significant influence on the food security of certain importing countries. This finding substantiated Burkholz and Schweitzer‘s highlight on the evolving role of countries as intermediaries in trade [16]. On the other hand, some African, Latin American and Caribbean countries were major importers with varying levels of vulnerability, as previous work has revealed [44], reflecting potential challenges in ensuring import security.

The regression analysis’s most significant finding was the notable impact of the constraint indicator on the PoU. Constraint, an indicator of structural hole scarcity, elucidates the inherent structural disadvantages of linking to strong partners who exhibit lower dependence on specific connections. The PoU serves as a critical indicator of food insecurity. To determine the impact of structural disadvantages on a country’s food insecurity, a regression model was adopted with cross-country panel data from 180 countries from 2001 to 2021. The findings suggest that a reduction of 0.36 in the constraint value could result in a one percentage point decrease in the PoU. Importantly, the results emphasized that the structural characteristics of import connections, rather than import dependency alone, play a major role in determining the prevalence of undernourishment. Furthermore, the heterogeneity analysis reveals regional variations in the impact of centrality and constraint on food insecurity. Areas heavily reliant on rice as a staple, such as Asia, Africa, and Latin America, experience more pronounced effects from these various factors. This finding aligns with Su et al.’s findings [45], indicating that economic policy uncertainty and foreign trade dependence have diverse inhibitory effects on food security for developed and developing countries.

This paper has certain limitations. First, it does not analyze the causes of specific risk formation and specific solutions from a more macroscopic perspective. Second, only the impacts of trade quantity and trade structure are discussed; the risks arising from price, export policy, transportation, and other factors were not considered in the network analysis nor were the trade frictions caused by political factors.

## 5. Conclusions

This study employed network analysis methods to intricately depict the characteristics, including scale, density, and connectivity, of the international rice trade network as well as its dynamic changes over the past three decades. The analysis of the development patterns indicated that the overall efficiency and stability of the rice trade network have improved, while this stability primarily stems from the increased power of core countries. Some network structural features were identified that may impact trade resilience and food security. Furthermore, utilizing network analysis indicators as crucial independent variables, a panel data model was established to regress against food security indicators. Through the causal inference, this study discovered that a country’s structural hole disadvantage within its trade network has a significant impact on food insecurity, when other conditions are unchanged. These results have been validated through various robustness and endogeneity tests.

What sets this study apart is its innovative combination of network analysis and the use of a panel data regression model, building the causality relationship between the network features and food security. The implication from the findings for enhancing food security is to decrease the constraint values in the trade network. Burt’s concept of constraint measures how much an individual’s actions are limited by its position in the network. A high constraint value suggests limited trading partners, potentially limiting product and resource diversity through trade. This vulnerability may increase susceptibility to economic shocks, reducing the ability to absorb and recover from disruptions. Moreover, countries with high constraint may face challenges in negotiating favorable trade agreements or navigating complex trade policies. Reducing constraint values, i.e., improving structural holes, could be achieved by establishing connections with partner countries who have less connections or are in a weak position in the network. For instance, as rice production increases in Sri Lanka, establishing trade connections with formerly weak exporting countries like this can prove beneficial for net-importing nations. In conclusion, understanding the evolving dynamics of the rice trade network provides valuable insights for policymakers aiming to navigate the complexities of global food trade. By adopting proactive and collaborative measures, countries can contribute to a more resilient and sustainable rice trade system in the face of evolving global challenges.

Future research could focus on the following: (1) understanding the mechanisms of improving structural holes; (2) measuring the impact of intermediary countries on global food security; (3) combining other economic and social elements to examine the causality within the network analysis framework.

## Figures and Tables

**Table 1:** Descriptions of network-level measures.

Classification	Measure	Description
Network Size	Number of Ties	Number of connections of each node
	Avg Degree	Average number of connections of each node
	Density	Proportion of the actual existing trade links to all possible trade links
	Diameter	Maximum geodesic distance
	Sum Strength	Sum of the trade volume of all the ties
Network Centralization	Deg Centralization	Degree of distribution in the network compared to a perfectly centralized network
	Out-Centralization	Degree of centralization in the export direction
	In-Centralization	Degree of centralization in the import direction
	Indeg H-Index	Calculated by the largest number x, such that there are x vertices of the in-degree of at least x. It helps identify the most influential nodes based on the number of incoming connections
	K-Core Index	Subgraph where all nodes have at least k connections to other nodes in the subgraph
	Indeg Corr	In-degree correlation or assortativity of the network
	Outdeg Corr	Out-degree correlation or assortativity of the network
	Maximum Strength	Highest trade volume on the connections
	Avg Strength	Average trade volume on the connections
Network Connectivity	Components	Sets of nodes that are connected to each other but not to nodes outside the set
	Component Ratio	Indicates how many distinct disconnected clusters or subgraphs exist within the network
	Compactness	Mean of all the reciprocal distances—it is a measure of how closely knit a network is
	Breadth	Equals one minus the compactness.
	Connectedness	Extent to which all nodes in the network are reachable from any other node
	Fragmentation	Complement of connectedness (1-Connectedness), representing the proportion of vertex pairs that are unreachable. It measures how much a network breaks into disconnected components
	Transitivity/Closure	Calculated by the number of nonvacuous transitive triples divided by number of paths of length 2. It reflects the degree of clustering in the network
	Avg Distance	Average geodesic distance among reachable pairs
	Prop within 3	Calculates the proportion of nodes within three steps from each node
	SD Distance	Standard deviation of the geodesic distances among reachable pairs: a lower SD distance indicates a more homogeneous network in terms of how close or far nodes are from each other
	Wiener Index	Sum of the geodesic distances between all pairs of nodes. It is a measure of the total closeness or connectedness of the network
	Dependency Sum	Calculated by the sum of all the geodesic distance minus n(n-1). It measures the extent to which nodes in the network are dependent on one another
	Nulls	Proportion of dyads (pairs of nodes) that have no links
	Mutuals	Proportion of dyads that have reciprocated links
	Asymmetrics	Proportion of dyads that have an unreciprocated links and assess the asymmetry in relationships between nodes
	Arc Reciprocity	Number of reciprocated arcs (connections between nodes) divided by the total number of arcs
	Dyad Reciprocity	Number of reciprocated dyads divided by the total number of dyads

**Table 2:** Whole network measures of the global rice trade from 1986 to 2021.

Classification	Measure	1986	2021	Change from 1986 to 2021	Values from 1986 to 2021	
					Mean	Std. Dev.
Network Size	Number of Ties	426	2149	404.46%	1354.278	604.202
	Avg Degree	2.029	10.233	404.34%	6.449	2.877
	Density	1.00%	4.90%	3.9 pp	3.10%	1.40%
	Diameter	7	6	−14.29%	5.972	0.609
	Sum Strength	5,863,727	50,943,092	768.78%	25,553,666	14,421,910
Network Centralization	Deg Centralization	25.20%	69.90%	44.7 pp	47.60%	14.50%
	Out−Centralization	23.10%	73.40%	50.3 pp	49.20%	16.40%
	In−Centralization	7.20%	17.20%	10 pp	12.30%	3.50%
	Indeg H−Index	8	20	150.00%	15.056	4.105
	K−core index	7	18	157.14%	13.333	3.84
	Indeg Corr	10.90%	12.40%	1.5 pp	8.50%	1.70%
	Outdeg Corr	21.60%	30.60%	9 pp	27.80%	3.20%
	Maximum Strength	856,952	2,479,740	189.37%	1,742,748	1,075,720
	Avg Strength	13,764.62	23,705.49	72.22%	17,743	3368
Network Connectivity: Components, Distance, and Reciprocity	Components	180	117	−35.00%	143.278	23.911
	Component Ratio	85.60%	55.50%	−30.1 pp	68.10%	11.40%
	Compactness	4.50%	20.80%	16.3 pp	13.50%	5.70%
	Breadth	95.50%	79.20%	−16.3 pp	86.50%	5.70%
	Connectedness	10.50%	42.50%	32 pp	29.00%	11.80%
	Fragmentation	89.50%	57.50%	−32 pp	71.00%	11.80%
	Transitivity/Closure	22.50%	34.70%	12.2 pp	31.90%	4.40%
	Avg Distance	2.788	2.334	−16.28%	2.538	0.119
	Prop Within 3	8.10%	39.60%	31.5 pp	25.20%	10.80%
	SD Distance	1.049	0.803	−23.45%	0.914	0.088
	Wiener Index	12,895	43,509	237.41%	31,884	12,427
	Dependency Sum	8269	24,871	200.77%	19,170	7277
	Nulls	98.20%	91.30%	−7.03 pp	94.42%	2.40%
	Mutuals	0.10%	1.10%	1 pp	0.60%	0.30%
	Asymmetrics	1.70%	7.70%	6 pp	5.00%	2.10%
	Arc Reciprocity	13.10%	21.90%	8.8 pp	18.10%	2.60%
	Dyad Reciprocity	7.00%	12.30%	5.3 pp	10.00%	1.60%

Notes: The number of nodes remained fixed at 210 from 1986 to 2021 to ensure comparability across the years. Except for sum strength, maximum strength, and avg strength, other indicators were calculated using binary data. Calculations were performed using Ucinet 6.75. The mean and std. dev. were calculated based on 36 sample values from 1986 to 2021, and “pp” stands for percentage point.

**Table 3:** List of the top five countries by degree centrality, closeness centrality, and betweenness centrality.

Rank	Outdegree					Outstrength				
	1986	1992	2008	2014	2021	1986	1992	2008	2014	2021
1	USA	PAK	IND	IND	IND	THA	THA	THA	IND	IND
2	FRA	CHN	PAK	PAK	PAK	USA	VNM	USA	THA	THA
3	THA	IND	THA	THA	THA	URY	USA	IND	VNM	PAK
4	IND	USA	USA	USA	USA	ARG	PAK	VNM	PAK	VNM
5	BLX	THA	CHN	ITA	ITA	ITA	CHN	PAK	USA	USA
**Rank**	**Outcloseness**					**Betweenness**				
	**1986**	**1992**	**2008**	**2014**	**2021**	**1986**	**1992**	**2008**	**2014**	**2021**
1	USA	PAK	IND	IND	IND	USA	USA	USA	USA	USA
2	THA	USA	PAK	PAK	PAK	FRA	CHN	PAK	PAK	IND
3	IND	IND	THA	THA	THA	BLX	DEU	ITA	NER	DEU
4	ITA	THA	USA	USA	USA	CAN	BLX	FRA	FRA	FRA
5	FRA	CHN	CHN	VNM	ITA	IND	FRA	DEU	ZAF	ZAF

Notes: Degree centrality is measured by both the number of trade connections (degree) and the trade volume (strength). The top countries are presented in terms of only outdegree/strength centrality and outcloseness centrality because the influence in the export direction is much more concentrated than that in the import direction.

**Table 4:** Descriptive statistics for variables in the main model.

Variables	Mean	Median	Maximum	Minimum	Std. Dev.	Observations	Cross Sections
PoU (*Y*_1_)	9.730	5.700	67.800	0.000	11.414	3578	180
Outdegree	9.623	1.000	158.000	0.000	20.950	3578	180
Indegree	9.589	8.000	48.000	0.000	6.794	3578	180
Constraint	0.847	0.796	2.000	0.053	0.417	3578	180
lnGDP	8.427	8.421	11.804	4.464	1.520	3578	180
CIDR	24.969	29.300	100.000	−654.900	65.565	3578	180

**Table 5:** The egression results for the main model on dependent variable PoU (*Y_1_*) and on alternative food security measures (*Y_e_*, *Y_GFSI_*).

Variables	*Y*_1_						*Ye*	*YGFSI*
	(1)	(2)	(3)	(4)	(5)	(6)	(7)	(8)
Outdegree	−0.0875 *** (0.0090)			−0.0223 ** (0.0102)	−0.00955 (0.0075)	−0.0056 (0.0075)	−0.0049 (0.0101)	0.0248 *** (0.0068)
Indegree		−0.4071 *** (0.0282)		−0.2715 *** (0.0335)	0.0802 *** (0.0254)	0.0836 *** (0.0252)	0.1328 *** (0.0346)	−0.0585 ** (0.0234)
Constraint			5.9956 *** (0.4464)	3.2319 *** (0.5493)	3.1268 *** (0.4025)	2.7464 *** (0.4035)	−4.2332 *** (0.5630)	−1.6367 *** (0.5261)
lnGDP					−5.2677 *** (0.0951)	−5.2847 *** (0.0945)	6.1921 *** (0.1326)	7.3275 *** (0.1112)
CIDR						0.0146 *** (0.0021)	−0.0148 *** (0.0029)	−0.0047 ** (0.0022)
C	10.5727 *** (0.2064)	13.6344 *** (0.3273)	4.6497 *** (0.4212)	9.8103 *** (0.7091)	50.7964 *** (0.9043)	50.8285 *** (0.8983)	70.4679 *** (1.1104)	−1.0727 (1.1104)
Period Fixed	yes	yes	yes	yes	yes	yes	yes	yes
Adjusted R2	0.0348	0.0639	0.0568	0.0775	0.5047	0.5112	0.4796	0.8410
Root Mean Square Error (RMSE)	11.1792	11.0092	11.0506	10.9260	8.0048	7.9507	10.7679	4.9141
Akaike Information Criterion (AIC)	7.6783	7.6476	7.6551	7.6336	7.0119	6.9989	7.6061	6.0503
N	3578	3578	3578	3578	3578	3578	3438	1063

Notes: The cross-section is not fixed. Standard errors are given in parentheses. ** *p* < 0.05, *** *p* < 0.01.

**Table 6:** IV Regression results.

Variables	*Y*_1_			
	(9)	(10)	(11)	(12)
Foutdegree	−0.5405 *** (0.0227)			0.0117 (0.0232)
Findegree		−1.1711 *** (0.0429)		0.0836 * (0.0449)
Fconstraint			21.7579 *** (0.8111)	10.2260 *** (0.8570)
*lnGDP*				−4.9552 *** (0.1066)
*CIDR*				0.0055 ** (0.0023)
C	14.9884 *** (0.2823)	20.9377 *** (0.4451)	−8.5592 *** (0.7035)	41.8448 *** (1.2393)
Period Fixed	yes	yes	yes	yes
Adjusted R2	0.1457	0.1809	0.1758	0.5260
RMSE	10.5170	10.2980	10.3305	7.8294
AIC	7.5562	7.5141	7.5204	6.9682
N	3578	3578	3578	3578

Notes: * *p* < 0.10, ** *p*<0.05, *** *p* < 0.01.

**Table 7:** Regression results based on geographical regional groups.

Variables	*Y*_1_									
	(13) Asia	(14) Europe	(15) Africa	(16) Americas	(17) Oceania	(18) Latin America and the Caribbean	(19) Sub-Saharan Africa	(20) Least Developed Countries	(21) Low-Income Food Deficit Countries	(22) Net Food Importing Developing Countries
Outdegree	0.0404 *** (0.0104)	0.0141 * (0.0075)	−0.0424 (0.042)	−0.0162 (0.0197)	−0.0622 (0.0455)	0.0151 (0.0214)	0.01270 (0.0098)	−0.0092 (0.0129)	−0.017 (0.012)	0.0059 (0.0097)
Indegree	−0.1565 ** (0.0608)	0.0013 (0.0212)	−0.1857 ** (0.0827)	0.3504 *** (0.0452)	−0.1575 (0.097)	0.1641 *** (0.0591)	0.0093 (0.0377)	0.0248 (0.0462)	0.0697 (0.0468)	0.0671 * (0.0372)
Constraint	5.1172 *** (0.7871)	1.7048 *** (0.3798)	1.838 (1.145)	1.1429 * (0.5928)	0.8969 (1.1536)	3.0079 *** (0.9492)	4.2521 *** (0.6564)	2.576 *** (0.7964)	2.8272 *** (0.8312)	3.0544 *** (0.5998)
lnGDP	−4.5185 *** (0.2035)	−1.3490 *** (0.1048)	−6.3877 ** (0.4061)	−8.968 *** (0.309)	−2.7963 *** (0.3983)	−4.9565 *** (0.2344)	−3.6216 *** (0.1532)	−4.5878 *** (0.2096)	−4.9471 *** (0.2169)	−5.0795 *** (0.1449)
CIDR	0.0252 *** (0.007)	0.0007 (0.0015)	0.0647** (0.0129)	0.0086 *** (0.0027)	0.0022 (0.0056)	0.0245 *** (0.0064)	0.0101 *** (0.0029)	0.0112 *** (0.0033)	0.0161 *** (0.0034)	0.0165 *** (0.003)
C	42.1185 *** (1.8082)	12.7839 *** (1.0867)	62.0539 ** (2.9525)	83.3066 *** (2.7234)	33.4787 *** (3.4923)	45.9739 *** (2.0703)	35.3609 *** (1.5306)	45.4294 *** (2.0144)	48.8116 *** (2.0918)	48.8239 *** (1.4183)
Period Fixed	yes	yes	yes	yes	yes	yes	yes	yes	yes	yes
Adjusted R2	0.5113	0.2235	0.2322	0.6023	0.4733	0.4331	0.4718	0.4548	0.5002	0.5250
RMSE	6.9060	2.8114	10.7551	5.6087	4.9136	8.4673	6.4570	7.2436	7.1866	7.4745
AIC	6.7598	4.9699	7.6404	6.3648	6.2900	7.1904	6.6249	6.8616	6.8468	6.8974
N	910	805	1005	664	194	649	918	819	806	1424
Cross-Sections	45	39	51	35	10	33	46	41	41	72

Notes: * *p* < 0.10, ** *p* < 0.05, *** *p* < 0.01. Sub-Saharan Africa including Sudan.

## Data Availability

Global Food Security Index, available at https://impact.economist.com/sustainability/project/food-security-index/ (accessed on 21 July 2023). Other data available at https://www.fao.org/faostat/en/#data/TM (accessed on 2 July 2023).

## Appendix A

**Table A1:** Country codes used in this paper.

**Country Code (M49)**	**Country**	**Code in This Paper**
4	Afghanistan	AFG
8	Albania	ALB
12	Algeria	DZA
24	Angola	AGO
28	Antigua and Barbuda	ATG
32	Argentina	ARG
51	Armenia	ARM
36	Australia	AUS
40	Austria	AUT
31	Azerbaijan	AZE
44	Bahamas	BHS
48	Bahrain	BHR
50	Bangladesh	BGD
52	Barbados	BRB
112	Belarus	BLR
56	Belgium	BEL
58	Belgium-Luxembourg	BLX
84	Belize	BLZ
204	Benin	BEN
64	Bhutan	BTN
68	Bolivia (Plurinational State of)	BOL
70	Bosnia and Herzegovina	BIH
72	Botswana	BWA
76	Brazil	BRA
96	Brunei Darussalam	BRN
100	Bulgaria	BGR
854	Burkina Faso	BFA
108	Burundi	BDI
132	Cabo Verde	CPV
116	Cambodia	KHM
120	Cameroon	CMR
124	Canada	CAN
140	Central African Republic	CAF
148	Chad	TCD
152	Chile	CHL
344	China, Hong Kong SAR	HKG
446	China, Macao SAR	MAC
156	China, mainland	CHN
158	China, Taiwan Province of	TWN
170	Colombia	COL
174	Comoros	COM
178	Congo	COG
184	Cook Islands	COK
188	Costa Rica	CRI
384	Côte d’Ivoire	CIV
191	Croatia	HRV
192	Cuba	CUB
196	Cyprus	CYP
203	Czechia	CZE
200	Czechoslovakia	CSK
408	Democratic People’s Republic of Korea	PRK
180	Democratic Republic of the Congo	ZAR
208	Denmark	DNK
262	Djibouti	DJI
212	Dominica	DMA
214	Dominican Republic	DOM
218	Ecuador	ECU
818	Egypt	EGY
222	El Salvador	SLV
226	Equatorial Guinea	GNQ
232	Eritrea	ERI
233	Estonia	EST
748	Eswatini	SWZ
231	Ethiopia	ETH
230	Ethiopia PDR	ETF
234	Faroe Islands	FRO
242	Fiji	FJI
246	Finland	FIN
250	France	FRA
254	French Guiana	GUF
258	French Polynesia	PYF
266	Gabon	GAB
270	Gambia	GMB
268	Georgia	GEO
276	Germany	DEU
288	Ghana	GHA
300	Greece	GRC
308	Grenada	GRD
312	Guadeloupe	GLP
320	Guatemala	GTM
324	Guinea	GIN
624	Guinea-Bissau	GNB
328	Guyana	GUY
332	Haiti	HTI
340	Honduras	HND
348	Hungary	HUN
352	Iceland	ISL
356	India	IND
360	Indonesia	IDN
364	Iran (Islamic Republic of)	IRN
368	Iraq	IRQ
372	Ireland	IRL
376	Israel	ISR
380	Italy	ITA
388	Jamaica	JAM
392	Japan	JPN
400	Jordan	JOR
398	Kazakhstan	KAZ
404	Kenya	KEN
296	Kiribati	KIR
414	Kuwait	KWT
417	Kyrgyzstan	KGZ
418	Lao People’s Democratic Republic	LAO
428	Latvia	LVA
422	Lebanon	LBN
426	Lesotho	LSO
430	Liberia	LBR
434	Libya	LBY
440	Lithuania	LTU
442	Luxembourg	LUX
450	Madagascar	MDG
454	Malawi	MWI
458	Malaysia	MYS
462	Maldives	MDV
466	Mali	MLI
470	Malta	MLT
584	Marshall Islands	MHL
474	Martinique	MTQ
478	Mauritania	MRT
480	Mauritius	MUS
484	Mexico	MEX
583	Micronesia (Federated States of)	FSM
496	Mongolia	MNG
499	Montenegro	MONT
504	Morocco	MAR
508	Mozambique	MOZ
104	Myanmar	MMR
516	Namibia	NAM
520	Nauru	NRU
524	Nepal	NPL
528	Netherlands (Kingdom of the)	NLD
540	New Caledonia	NCL
554	New Zealand	NZL
558	Nicaragua	NIC
562	Niger	NER
566	Nigeria	NGA
570	Niue	NIU
807	North Macedonia	MKD
578	Norway	NOR
512	Oman	OMN
586	Pakistan	PAK
275	Palestine	PALE
591	Panama	PAN
598	Papua New Guinea	PNG
600	Paraguay	PRY
604	Peru	PER
608	Philippines	PHL
616	Poland	POL
620	Portugal	PRT
630	Puerto Rico	PRI
634	Qatar	QAT
410	Republic of Korea	KOR
498	Republic of Moldova	MDA
638	Réunion	REU
642	Romania	ROM
643	Russian Federation	RUS
646	Rwanda	RWA
659	Saint Kitts and Nevis	KNA
662	Saint Lucia	LCA
670	Saint Vincent and the Grenadines	VCT
882	Samoa	WSM
678	Sao Tome and Principe	STP
682	Saudi Arabia	SAU
686	Senegal	SEN
688	Serbia	SERI
891	Serbia and Montenegro	SER
690	Seychelles	SYC
694	Sierra Leone	SLE
702	Singapore	SGP
703	Slovakia	SVK
705	Slovenia	SVN
90	Solomon Islands	SLB
706	Somalia	SOM
710	South Africa	ZAF
728	South Sudan	SSUD
724	Spain	ESP
144	Sri Lanka	LKA
729	Sudan	SUDA
736	Sudan (former)	SDN
740	Suriname	SUR
752	Sweden	SWE
756	Switzerland	CHE
760	Syrian Arab Republic	SYR
762	Tajikistan	TJK
764	Thailand	THA
626	Timor-Leste	TMP
768	Togo	TGO
772	Tokelau	TKL
776	Tonga	TON
780	Trinidad and Tobago	TTO
788	Tunisia	TUN
792	Türkiye	TUR
795	Turkmenistan	TKM
798	Tuvalu	TUV
800	Uganda	UGA
804	Ukraine	UKR
784	United Arab Emirates	ARE
826	United Kingdom of Great Britain and Northern Ireland	GBR
834	United Republic of Tanzania	TZA
840	United States of America	USA
858	Uruguay	URY
810	USSR	SVU
860	Uzbekistan	UZB
548	Vanuatu	VUT
862	Venezuela (Bolivarian Republic of)	VEN
704	Viet Nam	VNM
887	Yemen	YEM
890	Yugoslav SFR	YUG
894	Zambia	ZMB
716	Zimbabwe	ZWE

## References

[1] Muthayya S., Sugimoto J.D., Montgomery S., Maberly G.F. (2014). An overview of global rice production, supply, trade, and consumption. Ann. N. Y. Acad. Sci..

[2] Jayne T.S. (1993). Sources and Effects of Instability in the World Rice Market.

[3] Nguyen H.T.M., Do H., Kay A., Kompas T. (2020). Rice policy in a transitional economy: Balancing the social and political objectives. Food Secur..

[4] World Bank Food Security Update 27 July 2023. https://thedocs.worldbank.org/en/doc/40ebbf38f5a6b68bfc11e5273e1405d4-0090012022/food-security-update.

[5] Wailes E.J. (2005). Rice: Global trade, protectionist policies, and the impact of trade liberalization. Global Agricultural Trade and Developing Countries.

[6] Anderson K. (2022). Trade-related food policies in a more volatile climate and trade environment. Food Policy.

[7] Zeigler R. (2014). Perspective: Time to unleash rice. Nature.

[8] Zhang C., Yang Y., Feng Z., Xiao C., Lang T., Du W., Liu Y. (2021). Risk of global external cereals supply under the background of the covid-19 pandemic: Based on the perspective of trade network. Foods.

[9] Van Berkum S. (2021). How trade can drive inclusive and sustainable food system outcomes in food deficit low-income countries. Food Secur..

[10] D’Amour C.B., Anderson W. (2020). International trade and the stability of food supplies in the Global South. Environ. Res. Lett..

[11] Wheeler T., von Braun J. (2013). Climate Change Impacts on Global Food Security. Science.

[12] Hubbard L.J., Hubbard C. (2013). Food security in the United Kingdom: External supply risks. Food Policy.

[13] Fair K.R., Bauch C.T., Anand M. (2017). Dynamics of the Global Wheat Trade Network and Resilience to Shocks. Sci. Rep..

[14] Tamea S., Laio F., Ridolfi L. (2016). Global effects of local food-production crises: A virtual water perspective. Sci. Rep..

[15] Puma M.J., Bose S., Chon S.Y., Cook B.I. (2015). Assessing the evolving fragility of the global food system. Environ. Res. Lett..

[16] Burkholz R., Schweitzer F. (2019). International crop trade networks: The impact of shocks and cascades. Environ. Res. Lett..

[17] Scott J. (2011). Social network analysis: Developments, advances, and prospects. Soc. Netw. Anal. Min..

[18] Jafari Y., Engemann H., Zimmermann A. (2023). Food trade and regional trade agreements—A network perspective. Food Policy.

[19] Gutiérrez-Moya E., Adenso-Díaz B., Lozano S. (2021). Analysis and vulnerability of the international wheat trade network. Food Secur..

[20] Krijkamp A.R., Knoben J., Oerlemans L.A.G., Leenders R.T.A.J. (2021). An ace in the hole: The effects of (in)accurately observed structural holes on organizational reputation positions in whole networks. J. Bus. Res..

[21] Raj S., Brinkley C., Ulimwengu J. (2022). Connected and extracted: Understanding how centrality in the global wheat supply chain affects global hunger using a network approach. PLoS ONE.

[22] Wu F., Guclu H. (2013). Global Maize Trade and Food Security: Implications from a Social Network Model. Risk Anal..

[23] Sartori M., Schiavo S. (2015). Connected we stand: A network perspective on trade and global food security. Food Policy.

[24] Borgatti S.P., Everett M.G., Freeman L.C. (2002). Ucinet for Windows: Software for Social Network Analysis.

[25] Hanneman R., Riddle M. (2005). Introduction to Social Network Methods. https://LibreTexts.org.

[26] SageHogan B. (2008). Analyzing social networks. The Sage Handbook of Online Research Methods.

[27] Burt R.S. (1992). Structural Holes: The Social Structure of Competition.

[28] Graham A.V., McLevey J., Browne P., Crick T. (2022). Structural diversity is a poor proxy for information diversity: Evidence from 25 scientific fields. Soc. Netw..

[29] Burt R.S. (2004). Structural holes and good ideas. Am. J. Sociol..

[30] FAO, IFAD, UNICEF, WFP, WHO (2022). The State of Food Security and Nutrition in the World 2022. Repurposing Food and Agricultural Policies to Make Healthy Diets More Affordable.

[31] Lazaroiu G., Andronie M., Uţă C., Hurloiu I. (2019). Trust Management in Organic Agriculture: Sustainable Consumption Behavior, Environmentally Conscious Purchase Intention, and Healthy Food Choices. Front. Public Health.

[32] Majerova J., Sroka W., Krizanova A., Gajanova L., Lazaroiu G., Nadanyiova M. (2020). Sustainable brand management of alimentary goods. Sustainability.

[33] Zhao L., Poh C.N., Wu J., Zhao X., He Y., Yang H. (2022). Effects of electrolysed water combined with ultrasound on inactivation kinetics and metabolite profiles of Escherichia coli biofilms on food contact surface. Innov. Food Sci. Emerg. Technol..

[34] Chai Y., Yu Y., Zhu H., Li Z., Dong H., Yang H. (2023). Identification of common buckwheat (Fagopyrum esculentum Moench) adulterated in Tartary buckwheat (*Fagopyrum tataricum* (L.) Gaertn) flour based on near-infrared spectroscopy and chemometrics. Curr. Res. Food Sci..

[35] FAO, FAOSTAT (2023). Trade: Detailed Trade Matrix. https://www.fao.org/faostat/en/#data/TM.

[36] Cramer G.L., Wailes E.J., Shui S. (1993). Impacts of Liberalizing Trade in the World Rice Market. Am. J. Agric. Econ..

[37] Akhter S. (2017). Market integration between surplus and deficit rice markets during global food crisis period. Aust. J. Agric. Resour. Econ..

[38] Childs N. (2014). Rice Outlook. https://www.ers.usda.gov/webdocs/outlooks/38859/46657_rcs-14e.pdf?v=8688.1.

[39] FAO, IFAD, UNICEF, WFP, WHO (2023). The State of Food Security and Nutrition in the World 2023. Urbanization, Agrifood Systems Transformation and Healthy Diets Across the Rural–Urban Continuum.

[40] Dupas M.C., Halloy J., Chatzimpiros P. (2019). Time dynamics and invariant subnetwork structures in the world cereals trade network. PLoS ONE.

[41] Wang J., Dai C. (2021). Evolution of global food trade patterns and its implications for food security based on complex network analysis. Foods.

[42] FAO (2022). The State of Agricultural Commodity Markets 2022. The Geography of Food and Agricultural Trade: Policy Approaches for Sustainable Development.

[43] Debnath D., Babu S., Ghosh P., Helmar M. (2018). The impact of India’s food security policy on domestic and international rice market. J. Policy Model..

[44] Dithmer J., Abdulai A. (2017). Does trade openness contribute to food security? A dynamic panel analysis. Food Policy.

[45] Su F., Liu Y., Chen S.J., Fahad S. (2023). Towards the impact of economic policy uncertainty on food security: Introducing a comprehensive heterogeneous framework for assessment. J. Clean. Prod..

